# Comparison of Anterior only and Combined Anterior and Posterior Approach in Treating Lumbosacral Tuberculosis

**DOI:** 10.1038/s41598-019-53800-3

**Published:** 2019-12-06

**Authors:** Dong Sun, Ze-hua Zhang, Gang Mei, Tian-yong Hou, Yang Li, Jian-Zhong Xu, Fei Luo

**Affiliations:** Department of Orthopedics, Southwest Hospital, National & Regional United Engineering Laboratory of Tissue Engineering, The Third Military Medical University (Army Medical University) of PLA, Chongqing, 400038 China

**Keywords:** Clinical microbiology, Clinical microbiology, Tuberculosis, Tuberculosis

## Abstract

A combined anterior and posterior (AP) surgical approach is a popular treatment modality of lumbosacral tuberculosis, but it is often traumatic and complicated. The present study aims to find whether the anterior only approach with the ARCH plate system is less invasive than the AP approach in treating lumbosacral tuberculosis. The ARCH plate system is an innovative anatomic lumbosacral anterior multi-directional locking plate system which was devised with due consideration to the anatomic features of the lumbosacral spine and irregular destruction of involved vertebral endplates. In this retrospective study, 32 patients with lumbosacral tuberculosis underwent surgeries via either the anterior only approach (ARCH group, 18 patients) using the ARCH system or the conventional combined anterior and posterior approach (AP group, 14 patients). American Spinal Injury Association (ASIA) scores, Visual Analogue Scale (VAS) scores, Oswestry Disability Index (ODI), bone union status, ESR, CRP, intervertebral foraminal height between L5 and S1, the vertical height between the anterior upper edge of L5 and S1 vertebral body, lumbosacral angle, and the physiological lordosis of between L1 and S1 from both groups were recorded and compared. All patients were followed up for at least two years. The average duration of operation, blood loss, and length of hospital admission of the ARCH group (154.6 min, 361.1 ml&18.3days) was significantly smaller and shorter(p < 0.001, p < 0.001 & p = 0.008) that those of the AP group(465.5 min, 814.3 ml & 24.6days). The ODI score(p = 0.08, 0.471, 0.06, 0.07, 0.107), the VAS score(p = 0.099, 0.249, 0.073, 0.103, 0.273), the intervertebral foraminal height between L5 and S1(p = 0.826, 0.073, 0.085), L5-S1 height(p = 0.057, 0.234, 0.094), lumbosacral angle(p = 0.052, 0.242, 0.825), and L5-S1 lordosis(p = 0.146, 0.129, 0.053) of both groups showed no significant difference in any of the time points. The anterior only approach using the ARCH system is as effective as the combined anterior and posterior approach and is less traumatic in treating lumbosacral tuberculosis.

## Introduction

Tuberculosis (TB) is rapidly emerging driver of adult mortality in developing countries. Despite 2011 seeing over 1.4 million deaths attributable to tuberculosis, there is a scarcity of studies investigating the management of lumbosacral TB^1^. Lumbosacral TB is thought to make up 2–3% of spinal TB cases^2,3^. Factors related to lumbosacral spinal biomechanics, such as the natural lumbar lordosis, can influence the pattern of progression and the development of deformities^4^. Patients with lumbosacral TB often experience abscesses in the iliopsoas and pre-sacral regions, as well as destruction of anterior vertebral columns. A combined anterior and posterior approach is often necessary for achieving thorough debridement and to enable adequate internal fixation. However, this operation can be traumatic given the complex structure of the anatomy of the lumbosacral spine and the need for fixation stability^5–8^. Moreover, several existing internal fixation systems for anterior lumbosacral surgery are intended for degenerative diseases with intact vertebral endplates. Accordingly, there exists significant difficulty in using these systems for managing spinal TB patients, who often have irregular bony vertebral endplates^9–11^. In the present study, an innovative anatomic lumbosacral anterior multi-directional locking plate system (ARCH Plate, KangHui Medical Co., ChangZhou, China) was devised with due consideration to the anatomic features of the lumbosacral spine and irregular destruction of involved vertebral endplates. In our previous study, the features and methods of applying this system were elucidated^12^. In the present study, however, we aim to assess the clinical efficacy of this system by comparing the clinical results of the anterior only approach with the ARCH plate and the conventional combined approach.

## Patients and Methods

Study protocols were reviewed and approved by the Southwest Hospital Ethics Committee prior to the charts review and all methods were performed in accordance with the relevant guidelines and regulations. From June 2005 and April 2013, patients who underwent surgery for lumbosacral tuberculosis were included in the study. The diagnosis of lumbosacral TB was made upon the medical history, clinical manifestations, radiology, lab tests, and histology samples if possible. While patients who met the following criteria were excluded: previous pelvic or lumbosacral surgery; lumbosacral deformity caused by any other disease, such as adolescent scoliosis or ankylosing spondylitis; lesions confined to the posterior vertebral column and failure to finish the post-operative follow-ups. As a result, 32 patients with lumbosacral TB were included, and they were divided into two groups according to the surgery approach, which were: group ARCH (18 patients, anterior-only surgery) and group AP (14 patients, combined posterior–anterior surgery). Informed consent for patients were obtained prior to the surgery, stating they were aware that their clinical data would be used for scientific research.

**Figure 1:**
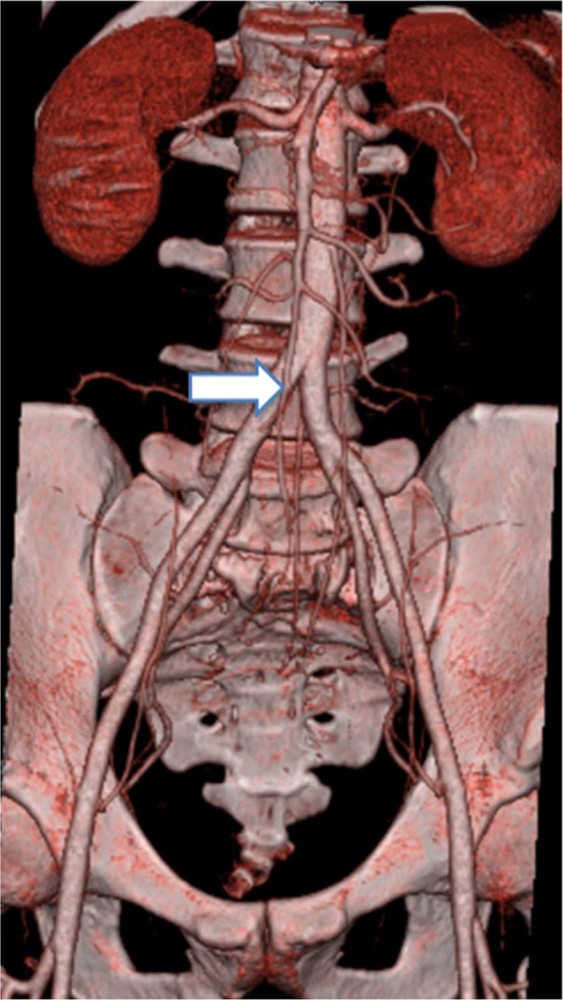
The pre-operative CTA demonstrates the level of the bifurcation of iliac vessels is suitable for internal fixation.

Preoperatively, all patients underwent X-ray, computed tomography (CT), digital subtraction angiography and magnetic resonance imaging (MRI) examinations were performed on all patients to reveal vertebrae damage and iliac vascular bifurcation (Fig. 1.), and an iliac vascular bifurcation that was too low (below the L5–S1 intervertebral space or the S1 vertebrae) had to undergo combined posterior–anterior surgery. Surgical indications included: (1) persistent lower back pain attributed to vertebral instability coexisting with pre-sacral or paraspinal abscess; (2) progressive neurological deficit; (3) presence of one or more local deformities likely to progress; and (4) patients unresponsive to anti-tuberculosis regimens.

### Surgical procedures

All patients in the ARCH group received general anesthesia with endotracheal intubation. All patients were positioned in the supine position and administrated with Cefazolin Sodium (0.5 g, intravenously) 30 minutes before the incision. Before incision, a C-arm fluoroscopy was performed to confirm level of spinal pathology. A left paramedian incision was first performed on the lower abdominal section, followed by anterior rectus sheath incision in order to displace the rectus abdominis to the right. Patients with previous abdominal surgery or pelvic inflammation leading to peritoneal adhesions underwent the transperitoneal approach. The right and left common iliac veins and arteries were both identified. A blunt dissection was performed between these two vessels to expose the pre-sacral abscess. Thorough debridement of sequestrum, granulated tissue, necrotic tissue, pus, psoas abscesses and necrotic discs was done. Afterwards, the dura sac and nerve roots were explored and subjected to at least three rinses with saline and hydrogen peroxide. The L5-S1 intervertebral space was distracted, and the lumbosacral angle and intervertebral height were restored. One or two appropriate size three cortical autologous iliac bone segments were utilized to fill the space. The ARCH plate was placed anteriorly to the L5 and S1 vertebral bodies, with care taken to properly positon the plate and direction of screws upon the residual boney structure. Wound closure was performed after drainage tubes were placed. Extracted specimens were marked for drug sensitivity tests and pathology.

With regards to patients in the AP group, they were treated in the same manner anteriorly as those patients of the ARCH group, except for ARCH plate implantation. Afterwards, the patient was rotated to prone position, followed by sterilization and draping of the posterior lumbar region. Exposure of the posterior spinal elements, including lamina, transverse processes and facet joints was done via extraperiosteal dissection, which involves a midline incision extending one vertebra above and below the involved segments with great care to preserve the posterior elements. Trans-pedicular screws were inserted into the affected vertebrae, if possible. In certain cases with extensive bony destruction of the first sacral vertebra, trans-pedicular screws were instead implanted in the ilium. Pre-bent rods were inserted bilaterally, and the kyphosis deformity was gradually corrected by compression and stretching of the internal fixation instrument. Drainage, wound closure, and resected specimens were performed similarly. Meanwhile, the amount of intra-operative blood loss and the duration of surgery were recorded in both groups.

### Post-operative care and follow-up protocols

Surgical sites were allowed to undergo postural drainage post-operatively, with drainage tubes removed once drainage volume was reduced to less than 30 ml. Patients were continued on 12 to 18-month regimens of oral HRE chemotherapy (6HREZ/6–18HRE), with cessation of pyrazinamide in six months. Brace-assisted ambulation was allowed after surgery for 6–8 weeks. Clinical and radiological assessments were performed at 3, 6, and 12 months after surgery and once a year thereafter.

### Measurement of clinical indices and statistical analysis

The following indices were recorded pre and post-operatively: American Spinal Injury Association (ASIA) scores, Oswestry Disability Index (ODI), the Visual Analogue Scale (VAS) scores; bone union status according to the Bridwell criteria^13^, ESR, CRP, intervertebral foraminal height between L5 and S1, the vertical height between the anterior upper edge of the L5 and S1 vertebral body, lumbosacral angle, and the physiological lordosis of between L1 and S1. The differences between the two groups in terms of duration of surgery (min), blood loss (ml), and hospitalization (days) were statistically analyzed with independently sampled t-tests. Cohorts that had non-normal data distributions were analyzed by a rank-sum test with the significance level set at 0.05. All data was analyzed with SPSS 22.0, with a p value of less than 0.05 considered statistically significant.

## Results

All patients were followed-up for at least 24 months. Follow-up time was 39.1 ± 12.0 months (range 24–67 months) for the ARCH group, while follow-up time was 40.7 ± 12.4 months (range 24–61 months) for the AP group. Patients of both groups received standard anti-tuberculous chemotherapy, including oral rifampin, isoniazid, ethambutol, and pyrazinamide for 2 to 12 weeks (average 4 weeks) before surgery and 12–18 (averaging 15 months) months post-operatively based on laboratory tests and clinical manifestations. In addition, all comorbidies, including anemia and hypoproteinemia, were properly treated before surgery.

Group ARCH included 18 consecutive patients who underwent single-stage anterior debridement and inter-body fusion with anatomic lumbosacral anterior multi-directional locking plates (ARCH Plate, KangHui Medical Co., ChangZhou, China). The mean age was 36 years old (range: 15–68 years). Pre-operatively, all patients exhibited various degrees of lower back pain, six experienced radicular pain of the lower extremities, and five presented with neurological deficits, including lower limb muscle weakness and sensory function impairment. The levels of the lesions were graded at L5-S1 (14 cases), and L5 (4 patients) presented with significant vertebral body damage and intervertebral space narrowing. Pre-sacral abscesses were observed in all 18 cases. Among them, one presented with iliac fossa abscesses and fistula formation, one with waist fistula, one with tuberculous pleuritis, and one with renal tuberculosis. The neurological American Spinal Injury Association (ASIA) scores were recorded preoperatively, with eight graded D and ten graded E.

Group AP included 14 consecutive patients who underwent anterior debridement, bone grafting, and posterior vertebral pedicle screw fixation. The mean age was 44 years (range of 18–72 years), with 6 males and 8 females. Pre-operatively, all patients exhibited various degrees of lower back pain, four experienced radicular pain of the lower extremities, and seven presented with neurological deficits, including lower limb muscle weakness and sensory function impairment. The levels of the lesions were graded as L5-S1 (12 cases) and L5 (1 case), and S1 (one case) presented with significant vertebral body damage and intervertebral space narrowing. Pre-sacral abscesses were observed in all 14 cases. The ASIA scores were recorded preoperatively, with five graded D and nine graded E.

Four patients from the ARCH group and six from the AP group experienced significant improvements in terms of radicular pain. The average operation time, blood loss, and hospital stay of the ARCH group was significantly shorter than those of the AP group (Table 1).

**Table 1:** Summary and comparison of clinical measurements between the two groups.

	ARCH group	AP group	P
Average operative time (min)	154.6 (110–220)	465.5 (185–885)	<0.001
Average blood loss (ml)	361.1 (200–800)	814.3 (400–2700)	<0.001
Hospital stay (days)	18.3 (11–33)	24.6 (17–38)	0.008

ARCH group = anatomic lumbosacral anterior multi-directional locking plate group.

AP group = Combined anterior and posterior group.

P < 0.05 was considered statistically significant.

**Figure 2:**
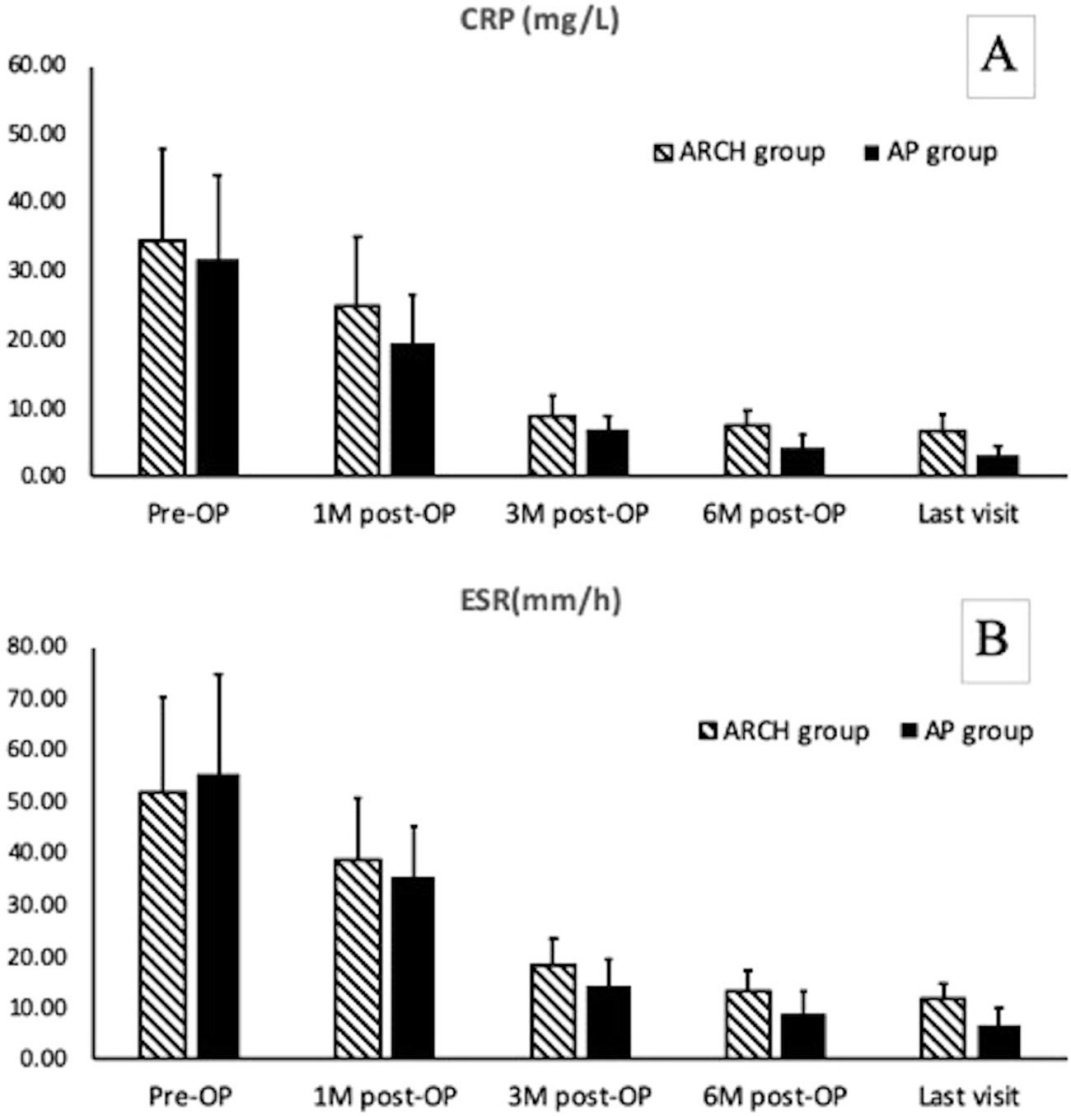
The chart demonstrates the CRP (**A**) and ESR (**B**) of pre-operation, post-operation, 1-month, 3-month, 6-month post-operation and last visit from both groups. In general, CRP and ESR of the two groups were improved significantly after surgery. (**A**) The average CRP(mg/L) of the ARCH group (18 patients) from all the time points (34.39 ± 13.45, 24.94 ± 10.03, 8.72 ± 3.03, 7.44 ± 2.23, 6.67 ± 2.45) showed no significant difference compared to the ones from the AP group(14patients), which were (31.8 ± 12.2, 19.4 ± 7.2, 6.9 ± 1.9, 4.2 ± 1.9, 3.1 ± 1.4). (**B**) The average ESRT(mm/h) of the ARCH group from all the time points (51.83 ± 18.41, 38.67 ± 12.12, 18.28 ± 5.3, 13.22 ± 3.84, 11.72 ± 2.99) showed no significant difference compared to the ones from the AP group, which were(55.36 ± 19.37, 35.43 ± 9.8, 14.43 ± 5.09, 9 ± 4.22, 6.79 ± 3.14).

The average preoperative ESR and CRP of the ARCH group were 55.4 mm/h (range of 1–94 mm/h) and 34.4 mg/L (range of 13–52 mg/L). For the AP group, they were 55.4 mm/h (range of 5–78 mm/h) and 31.8 mg/L (range of 16–50 mg/L). The ESR and CRP of most patients in both groups returned to normal three months after surgery (Fig. 2), with the exception of three patients. Two were from the ARCH group, whose ESR and CRP were 28 mm/h &11 mg/L, and 32 mm/h &18 mg/L, respectively. One was from the AP group, whose ESR and CRP were 24 mm/h &12 mg/L, respectively. Six months after surgery, the ESR and CRP of all patients returned to normal levels, which indicated the complete eradication of lesions in both groups (Fig. 2). Inter-group comparisons between the ARCH and AP groups in CRP and ESR at the above time points, however, showed no significant differences (CRP: p = 0.865, 0.1742, 0.969, 0.725, 0.627; ESR: p = 0.8741, 0.9082, 0.8279, 0.7667, 0.6322). In both groups, VAS and ODI score improved significantly post operation, yet comparison of the two above parameters between the two groups (Table 2) showed no significant differences in any of the time points, although the average ODI score of the AP group was slightly higher. With regards to neurological status, eight patients of the ARCH group and five patients of the AP group had neurological impairment before surgery, with an ASIA score of grade D. At the last follow-up, no evidence of neurological impairment in any of the patients was recorded.

**Table 2:** Summary and comparison of ODI & VAS between the two groups.

	ODI from group ARCH	ODI from group AP	*P*	VAS from group ARCH	VAS from group AP	*P*
Pre-operative	27.44 (16–50)	36.07 (19–58)	0.08	4.17 (2–8)	5.21 (3–8)	0.099
1-month post-op	19.50 (10–37)	21.64 (8–36)	0.471	3.00 (2–5)	3.50 (2–6)	0.249
3-month post-op	5.83 (4–16)	10.00 (4–27)	0.06	1.78 (1–3)	2.29 (1–4)	0.073
6-month post-op	3.94 (2–8)	6.07 (2–16)	0.07	1.17 (0–2)	1.50 (1–2)	0.103
Last follow up	2.50 (2–4)	3.43 (2–10)	0.107	0.72 (0–2)	1 (0–2)	0.273

ODI = Oswestry Disability Index.

VAS = Visual Analogue Scale.

P > 0.05 has no statistical significance.

**Figure 3:**
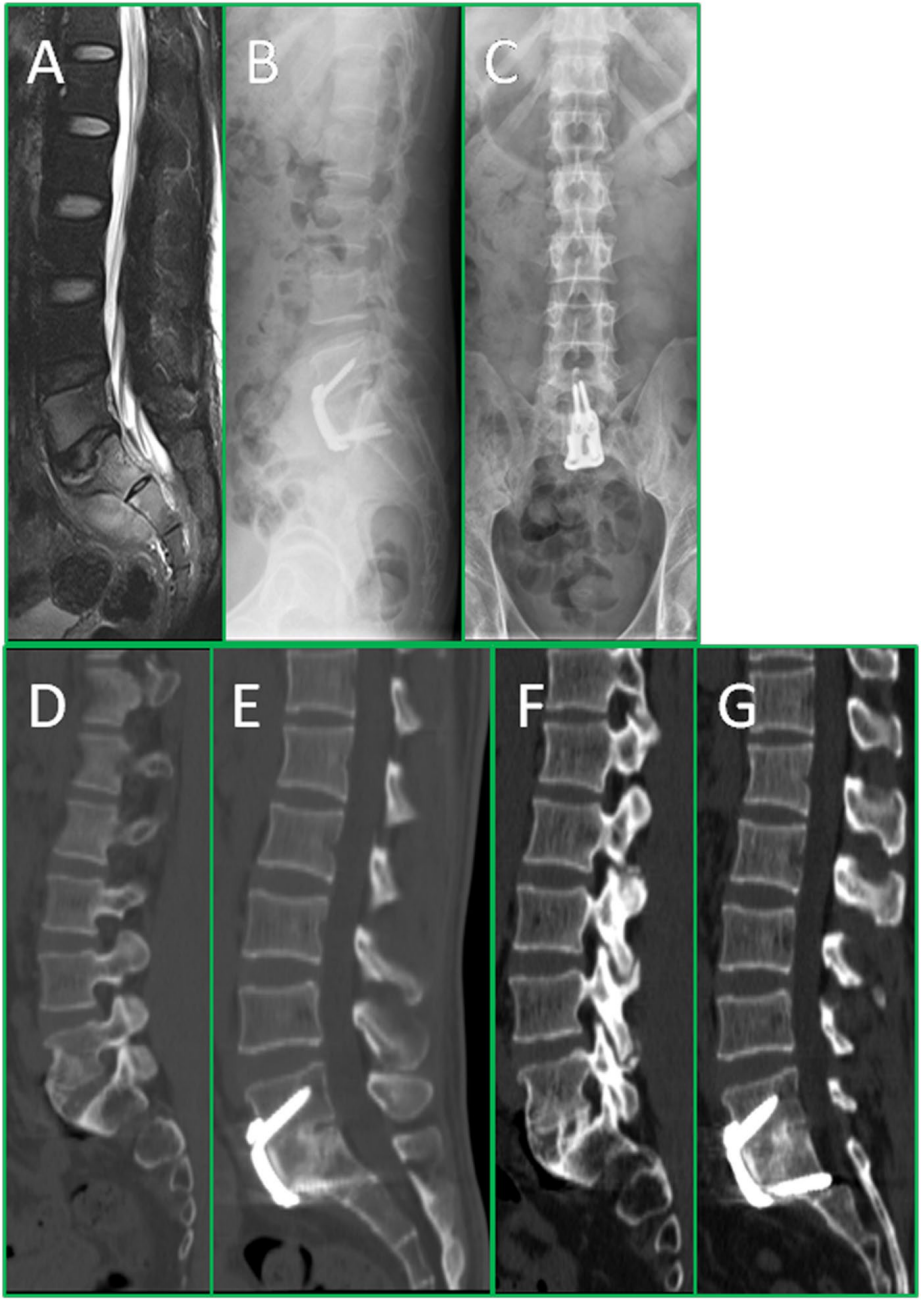
The series of radiographs show the radiological changes before and after treatment. This is a 35-year-old male patient, who had L5-S1 spinal TB with pre-sacral abscess, low back pain, and right lower limb radicular pain. The pre-operation MRI (**A**) shows bone destruction of L5 and S1 and pre-sacral cold abscess formation; The post-operative X-ray (**B**,**C**) confirmed the position of plate; The 12 months post-operative X-ray (**D**,**E**) shows definite bone fusion, while intervertebral height and the lumbosacral angle maintained satisfactory correction; The 24 months post-operative X-ray (**F**,**G**) reveals the ARCH plate still in position and good inter-body fusion of L5-S1, and the intervertebral height and lumbosacral angle were well maintained.

In the ARCH group, the intervertebral foraminal height between L5 and S1, L5-S1 height, lumbosacral angle, and lordosis of L5-S1 were significantly increased after surgery followed by a slight decrease without significance in the last follow-up (Table 3). The AP group followed similar patterns in three time points (Table 4). Inter-group comparisons between both ARCH and AP groups at three time points, however, showed no significant differences (Table 5). In the last follow-up, MRI scans revealed that several residual pre-sacral abscesses were absorbed without any local recurrence of tuberculosis (Fig. 3).

**Table 3:** The summary of radiological measurements of the ARCH group.

	Lumbosacral angle (°)	Physiological lordosis (L1-S1) (°)	Intervertebral foraminal height (L5-S1) (mm)	L5-S1 height(mm)
Preoperative	21.04 ± 4.69 (13.17–29.58)	29.15 ± 2.56 (24.55–32.26)	10.20 ± 1.23 (8.31–12.37)	34.77 ± 3.40 (29.06–40.23)
Postoperative	30.25 ± 1.81 (26.52–32.63)	40.29 ± 3.31 (33.47–43.69)	17.70 ± 1.14 (16.1–19.91)	42.90 ± 4.74 (37.28–55.91)
Last follow-up	27.12 ± 2.58 (22.79–30.84)	37.04 ± 2.37 (31.42–40.77)	16.38 ± 0.99 (15.29–17.99)	39.20 ± 4.02 (35.12–49.36)

**Table 4:** The summary of radiological measurements of the AP group.

	Lumbosacral angle (°)	Physiological lordosis (L1-S1) (°)	Intervertebral foraminal height (L5-S1) (mm)	L5-S1 height h(mm)
Preoperative	23.65 ± 1.19 (21.81–25.48)	27.81 ± 2.16 (24.17–31.27)	10.11 ± 1.10 (8.11–11.79)	34.50 ± 3.51 (30.32–42.19)
Postoperative	29.54 ± 1.41 (27.33–31.79)	39.72 ± 2.12 (35.48–42.79)	16.95 ± 1.11 (15.05–18.73)	42.47 ± 4.60 (37.64–53.77)
Last follow-up	27.29 ± 1.75 (23.56–29.82)	36.52 ± 2.36 (30.65–39.29)	15.76 ± 0.97 (14.02–18.14)	39.09 ± 3.41 (35.17–47.46)

**Table 5:** The intra- & inter- group comparison of radiological measurements.

Parameters	ARCH group	AP group	P***
Lumbosacral Angle			
Pre-operation	NA	NA	0.052
Immediate post-operation	p* < 0.001	p* < 0.001	0.242
Last follow-up	p** < 0.001	p** < 0.001	0.825
L5-S1 height			
Pre-operation	NA	NA	0.057
Immediate post-operation	p* < 0.001	p* < 0.001	0.234
Last follow-up	p** < 0.001	p** < 0.001	0.094
Lumbar Lordosis			
Pre-operation	NA	NA	0.146
Immediate post-operation	p* < 0.001	p* < 0.001	0.129
Last follow-up	p** < 0.001	p** < 0.001	0.053
L5-S1 intervertebral foramen height			
Pre-operation	NA	NA	0.826
Immediate post-operation	p* < 0.001	p* < 0.001	0.073
Last follow-up	p** < 0.001	p** < 0.001	0.085

NA = not available.

P* = p value of intra-group comparison between the pre-operation and immediate post operation.

P** = p value of intra-group comparison between the pre-operation and last follow-up.

P*** = p value of inter-group comparison between the ARCH group and AP group at all time points.

P > 0.05 has no statistical significance.

**Figure 4:**
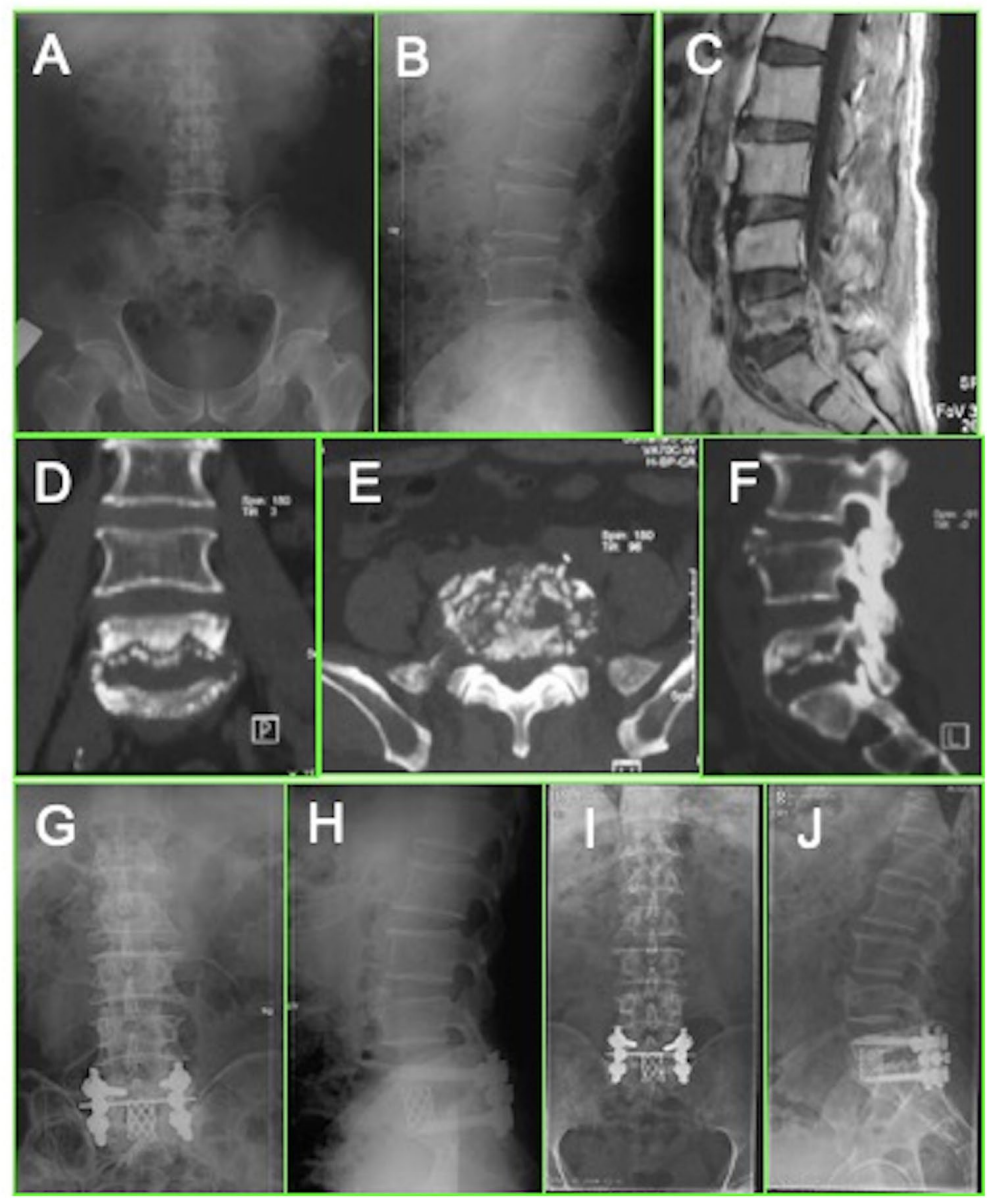
The series of radiographs show the radiological changes before and after treatment. This is a 49-year-old male patient, who had pulmonary tuberculosis and L5-S1 spinal TB with pre-sacral abscess and low back pain. The pre-operation X rays (**A**,**B**) and CT (**D**–**F**) show more than 2/3 bone destruction of L5 and pre-sacral cold abscess formation (**C**); The patient underwent anterior debridement and titanium strut with bone graft implantation followed by posterior fixation. The post-operative X-ray (**G**,**H**) confirmed the position of implants; The 12 months post-operative X-ray (**I**,**J**) showed definite bone fusion.

With regards bone fusion status, according to Bridwell criteria, all 32 patients achieved grade II bony fusion after 3–9 months and grade I bony fusion at 12 months after surgery (Fig. 3 & Fig. 4). No significant differences in fusion time or grade between the two groups were recorded. No implant loosening, or breakage were observed on the X-ray films at the last follow-ups in either group.

### Complications

In the ARCH group, two patients experienced peritoneal injuries intra-operatively, which were repaired immediately without the occurrence of further symptoms. One patient developed a fistula formation on the right side of the waist after operation, which was later diagnosed to be isoniazid and rifampicin-resistant tuberculosis. Accordingly, the patient’s drugs were changed, and the patient recovering two months after surgery. For either group, there was no report of erection dysfunction, reproductive femoral nerve irritation, or retrograde ejaculation after operation. In the last follow-up, there was no report of loosening or breakage of the implants, graft absorption or pseudarthrosis formation in either group.

## Discussion

Anterior only and two-stage combined anterior and posterior approaches comply with the requirements of stability reconstruction and can attain significantly positive outcomes in treating lumbosacral spinal TB. Historically, both approaches were reported in the treatment of L5-S1 tuberculosis. He and Xu^6^ performed single stage anterior debridement and anterior fixation on 13 patients with L5-S1 tuberculosis, all patients had bone fusion and pain relief 25 months post operation. At the same time, for patients who have significant vertebral damage (<1/3 affected vertebrae remaining) or whose iliac vascular bifurcation is not available for anterior fixation, the AP approach has been suggested^6,7^. There are three key justifications for carrying out a combined anterior and posterior approach: 1. The anatomical structure of the lumbosacral region is complex with limited range of exposure, especially in the presence of lesion-mediated tissue adhesion. Furthermore, this space is further minimized by the convoluted course of iliac vessels, as placement of internal fixation too close to a vessel risks the formation of an iliac pseudoaneurysm. 2. The need for efficient fixation implantation at the site of the TB lesion. 3. Current lumbosacral anterior internal fixation systems are unable to achieve adequate fixation efficiency. It can be difficult to maintain lumbar biomechanical constancy in the setting of local TB infections as lumbosacral TB often induces significant bony destruction of the anterior and middle spinal columns^6,14^.

The occurrence of vascular injury during stability reconstruction surgery and anterior internal fixation is estimated to be between 1.2% to 15.6%^5,8^. Inamasu *et al*. characterized an anatomical triangle anterior to the lumbosacral spine free from nerve trunks or major blood vessels, bordered superiorly by the sacral promontory and the iliac vessels on both sides^15^. Barrey *et al*. described an *in vivo* anatomic assessment using a preoperative 3D-CT on 146 patients to generate a vascular window^8^. A vascular window at L5-S1 was identified for the anterior L5-S1 disc. In other studies, anterior L5-S1 inter-body fusion and internal fixation were completed in this zone. According to our results, there exists several explicit factors for this zone: (i) the right common iliac artery and the left common iliac vein and their location; (ii) the angle of the right and left common iliac veins and their association with adjacent lumbosacral vertebrae; and (iii) override point width of the right common iliac vein and the left common iliac artery at differing points on the lumbosacral spine. Accordingly, the safe zone for implantation is constrained laterally by the external iliac vein and medially by the midline of the sacrum, which translates to space of approximately 30 mm in length and 24.5 mm (22–27 mm) in width. The ARCH plate was well engineered to fit such safe zone, based on these anatomic parameters^12^.

The main structures resisting anterior displacement of the L5 vertebrae (which is a risk, given that the L5 vertebrae bears the weight of the trunk) are the intervertebral space and the L5-S1 facet joint. A TB foci in the L5-S1 region may destroy the vertebral body and collapse the intervertebral disc, leaving patients at high risk of a decrease in lumbrosacral angle. The L5-S1 facet joint may be the only structure resisting L5-S1 sheer stress, and its tendency to detach may promote facet joint degeneration and lumbosacral pain. Surgical management of lumbosacral TB can restore vertebral body height by placing bone grafts in strategic locations to resist axial stress and local shearing, and by restabilizing the spine via internal fixation^7,16^. Nevertheless, anterior lumbosacral internal fixation systems, including SynFix-LR (Synthes, Solothurn, Switzerland)^17^, Trinica anterior lumbar plate system (Zimmor, Minneapolis, MN, USA)^18^ and Pyramid anterior lumbar plate (Sofamor,Menphis, TN, USA)^19^, were inadequate for surgical management of lumbosacral TB, given the presence of irregularities in the vertebral endplates. He and Xu demonstrated the safety and effectiveness of a single-stage anterior debridement and autograft fusion combined with two self-locking titanium anterior lumbosacral vertebrae plates (PACH; General Corp., Germany) in 13 patients with lumbosacral TB^6^. The average duration of operation, blood loss, length of hospital stay, and lumbosacral correction angle were 190 min, 410 mL, 15 days, and 5.1°, respectively. The authors brought forward several limitations of the PACH system, including: a vulnerable locking sheet preventing screw loosening, unstable parallelogram construction and complicated placement^12^. The ARCH plate was engineered based on preliminary anatomical research, and is narrow in the cephalic region while maintaining a wide triangle or trapezoid in the caudal region. The ARCH system also overcomes bony destruction of the endplates by incorporating multi-dimensional screws that are of increased length, inclination angles and are able to be re-positioned within the range of 5–20°. The screws are threaded with locking angle stability that prevents post-implantation retraction. Moreover, multi-directional screw technology enables the most ideal autologous iliac bone graft fitting onto irregular bone morphology, therefore acquiring maximal stability.

The present study was first to compare the outcomes of the anterior only approach with that of the combined anterior and posterior approaches. As indicated by our results, duration of operation, blood loss, and length of hospital stay of were significantly longer in the AP group compared to the ARCH group. All measured parameters in patients using the ARCH system were also superior to results obtained from other studies on the anterior approach^10,11^. Interestingly, the ARCH group achieved similar clinical outcomes to that of the AP group. The present study has demonstrated that the overall efficacy and efficiency of the anterior only approach with the ARCH system is superior to those of the combined approach.

Meanwhile, H. Zeng X *et al*. reported use of posterior only approach in treating lumbosacral tuberculosis, they concluded that for patients with confined anterior lesion, anterior debridement and posterior fixation should be considered first^14,20^. Now with the application of ARCH plate, patients who were not indicated for posterior only approach are likely to have one more choice.

However, the present study has several limitations including a small sample size and the fact that all patients presented with a disease affecting <4 vertebrae. Therefore, future research should consider a larger patient sample and a longer follow-up period.

## Conclusions

This retrospective study demonstrated the efficacy and efficiency of anterior debridement using the ARCH system in treating lumbosacral TB. It was found that such a method was as effective as that of the combined approach. Moreover, this method was found to be less traumatic and can provide similar clinical outcomes to those of the combined anterior and posterior approach.

## References

[1] Organization, W. H. Global Tuberculosis Report 2016. (World Health Organization 2016).

[2] Snider GL (1997). Tuberculosis then and now: a personal perspective on the last 50 years. Ann Intern Med.

[3] Rajasekaran S (1998). Tuberculous lesions of the lumbosacral region. A 15-year follow-up of patients treated by ambulant chemotherapy. Spine (Phila Pa 1976).

[4] Pun WK (1990). Tuberculosis of the lumbosacral junction. Long-term follow-up of 26 cases. J Bone Joint Surg Br.

[5] Wood KB, Devine J, Fischer D, Dettori JR, Janssen M (2010). Vascular injury in elective anterior lumbosacral surgery. Spine (Phila Pa 1976).

[6] He Q, Xu J (2012). Comparison between the antero-posterior and anterior approaches for treating L5-S1 vertebral tuberculosis. International orthopaedics.

[7] Kim DJ, Yun YH, Moon SH, Riew KD (2004). Posterior instrumentation using compressive laminar hooks and anterior interbody arthrodesis for the treatment of tuberculosis of the lower lumbar spine. Spine (Phila Pa 1976).

[8] Barrey C (2013). Vascular anatomy in the lumbar spine investigated by three-dimensional computed tomography angiography: the concept of vascular window. World neurosurgery.

[9] Zhang T (2017). One-Stage Anterolateral Debridement, Bone Grafting, and Internal Fixation for Treating Lumbosacral Tuberculosis. Asian spine journal.

[10] Wang WJ, Chen WK, Yan YG, Yao NZ, Wang C (2017). Application of anterior debridement and reconstruction with anatomical screw-plate fixation for lumbosacral tuberculosis: A 2-year-plus follow-up. Medicine.

[11] Li JH (2015). Surgical treatment of lumbosacral tuberculosis by one-stage debridement and anterior instrumentation with allograft through an extraperitoneal anterior approach. Journal of orthopaedic surgery and research.

[12] Luo F, Zhang ZH, Sun D, Xu JZ (2015). One-Stage Anterior Approach with Arch Plate to Treat Lumbosacral Tuberculosis. Orthopaedic surgery.

[13] Bridwell KH, Lenke LG, McEnery KW, Baldus C, Blanke K (1995). Anterior fresh frozen structural allografts in the thoracic and lumbar spine. Do they work if combined with posterior fusion and instrumentation in adult patients with kyphosis or anterior column defects?. Spine (Phila Pa 1976).

[14] Zeng H (2014). Posterior only versus combined posterior and anterior approaches in surgical management of lumbosacral tuberculosis with paraspinal abscess in adults. Eur J Trauma Emerg Surg.

[15] Inamasu J, Kim DH, Logan L (2005). Three-dimensional computed tomographic anatomy of the abdominal great vessels pertinent to L4-L5 anterior lumbar interbody fusion. Minimally invasive neurosurgery: MIN.

[16] Choi KC (2013). Biomechanical comparison of anterior lumbar interbody fusion: stand-alone interbody cage versus interbody cage with pedicle screw fixation–a finite element analysis. BMC musculoskeletal disorders.

[17] Schimmel JJ (2016). PEEK Cages in Lumbar Fusion: Mid-term Clinical Outcome and Radiologic Fusion. Clinical spine surgery.

[18] Lee, C. H., Hsu, C. C. & Huy, D. C. An optimization study of the screw orientation on the interfacial strength of the anterior lumbar plate system using neurogenetic algorithms and experimental validation. *Journal of biomechanical engineering* **136**, 10.1115/1.4028412 (2014). 10.1115/1.4028412 25162521

[19] Gerber M (2006). Biomechanical assessment of anterior lumbar interbody fusion with an anterior lumbosacral fixation screw-plate: comparison to stand-alone anterior lumbar interbody fusion and anterior lumbar interbody fusion with pedicle screws in an unstable human cadaver model. Spine (Phila Pa 1976).

[20] Zhang H (2015). Debridement, internal fixation, and reconstruction using titanium mesh for the surgical treatment of thoracic and lumbar spinal tuberculosis via a posterior-only approach: a 4-year follow-up of 28 patients. Journal of orthopaedic surgery and research.

